# Tumor edge-to-core transition promotes malignancy in primary-to-recurrent glioblastoma progression in a PLAGL1/CD109-mediated mechanism

**DOI:** 10.1093/noajnl/vdaa163

**Published:** 2020-11-27

**Authors:** Chaoxi Li, Hee Jin Cho, Daisuke Yamashita, Moaaz Abdelrashid, Qin Chen, Soniya Bastola, Gustavo Chagoya, Galal A Elsayed, Svetlana Komarova, Saya Ozaki, Yoshihiro Ohtsuka, Takeharu Kunieda, Harley I Kornblum, Toru Kondo, Do-Hyun Nam, Ichiro Nakano

**Affiliations:** 1 Department of Neurosurgery, Tongji Hospital, Tongji Medical College, Huazhong University of Science and Technology, Wuhan, China; 2 Research Institute for Future Medicine, Samsung Medical Center, Seoul, Republic of Korea; 3 Institute for Refractory Cancer Research, Samsung Medical Center, Sungkyunkwan University School of Medicine, Seoul, Republic of Korea; 4 Department of Neurosurgery, Ehime University, Japan; 5 Department of Neurology, University of Alabama at Birmingham, Birmingham, Alabama, USA; 6 Department of Geriatrics, Tongji Hospital, Tongji Medical College, Huazhong University of Science and Technology, Wuhan, China; 7 Intellectual and Developmental Disabilities Research Center, David Geffen School of Medicine at UCLA, Los Angeles, California, USA; 8 Department of Neurosurgery, University of Alabama at Birmingham, Birmingham, Alabama, USA; 9 Division of Stem Cell Biology, Institute for Genetic Medicine, Hokkaido University, Sapporo, Japan; 10 Department of Neurosurgery, Samsung Medical Center, Sungkyunkwan University School of Medicine, Seoul, Republic of Korea; 11 Research and Development Center for Precision Medicine, Tsukuba University, Tsukuba, Japan

**Keywords:** cancer stem cell, glioma stem cell, recurrence-initiating cell, spatial identity

## Abstract

**Background:**

Glioblastoma remains highly lethal due to its inevitable recurrence. Most of this recurrence is found locally, indicating that postsurgical tumor-initiating cells (TICs) accumulate at the tumor edge. These edge-TICs then generate local recurrence harboring new core lesions. Here, we investigated the clinical significance of the edge-to-core (E-to-C) signature generating glioblastoma recurrence and sought to identify its central mediators.

**Methods:**

First, we examined the association of E-to-C-related expression changes to patient outcome in matched primary and recurrent samples (*n* = 37). Specifically, we tested whether the combined decrease of the edge-TIC marker PROM1 (CD133) with the increase of the core-TIC marker CD109, representing E-to-C transition during the primary-to-recurrence progression, indicates poorer patient outcome. We then investigated the specific molecular mediators that trigger tumor recurrence driven by the E-to-C progression. Subsequently, the functional and translational significance of the identified molecule was validated with our patient-derived edge-TIC models in vitro and in vivo.

**Results:**

Patients exhibiting the CD133^low^/CD109^high^ signature upon recurrence representing E-to-C transition displayed a strong association with poorer progression-free survival and overall survival among all tested patients. Differential gene expression identified that *PLAGL1* was tightly correlated with the core TIC marker *CD109* and was linked to shorter patient survival. Experimentally, forced PLAGL1 overexpression enhanced, while its knockdown reduced, glioblastoma edge-derived tumor growth in vivo and subsequent mouse survival, suggesting its essential role in the E-to-C-mediated glioblastoma progression.

**Conclusions:**

E-to-C axis represents an ongoing lethal process in primary glioblastoma contributing to its recurrence, partly in a PLAGL1/CD109-mediated mechanism.

**Key Points**

- Edge-to-core (E-to-C) progression is a pathobiological process contributing to glioblastoma lethality.
- The CD133^low^/CD109^high^ signature is a novel prognostic molecular biomarker representing the E-to-C transition.
- PLAGL1 regulates the growth of tumor edge-located glioblastoma-initiating cells.

**Importance of the Study**

Very few studies have sought to longitudinally characterize the transition of molecular landscapes from primary to recurrent glioblastoma. Postsurgical edge-located TICs are presumably the predominant source of tumor recurrence, yet this cellular composition remains largely uncharacterized. This study evaluates the significance of glioblastoma edge-derived core (E-to-C) transition signature for lethal tumor recurrence with a paired primary-recurrent patient cohort. We elucidate a prognostically significant shift in molecular and cellular phenotypes associated with E-to-C by the CD133^low^/CD109^high^ dynamics. Moreover, our results provide a set of clinical and experimental evidence that the oncogenic transcription factor PLAGL1 represents an E-to-C determinant for glioblastoma development by the direct transcriptional regulation of the core TIC marker CD109.

Glioblastoma is an incurable universally lethal disease and characterized by inter- and intratumoral heterogeneity.^1–6^ Transcriptome-based subtyping of individual tumors is considered a milestone discovery of the past decade^7,8^; nonetheless, this molecular subtyping has yet to change clinical management, unlike other cancers that now have distinct treatment options instructed by particular genetic subtype information (eg, breast cancer, neuroblastoma).^9,10^ In sharp contrast to the accumulating experimental evidence for the mesenchymal shift of glioblastoma tumors being highly associated with a gain of malignancy and therapy resistance in various model systems, clinical data remain lacking to suggest that mesenchymal glioblastoma gains benefit from more extensive and/or different therapies. In addition, multiple independent large-scale studies have clarified that the transcriptomic subtype switch between primary and recurrent glioblastomas is simply a random event without any clear trend of 1 way or the other including toward the mesenchymal shift.^11^

Most glioblastomas recur within a few years as the main cause of its dismal prognosis in developed countries.^12^ A large degree of molecular difference between primary and recurrent tumors has been recognized by various OMICs analyses including deep sequencing, both with tumor tissues^13,14^ and at the single cell level.^15^ Since the brain tissues adjacent to surgical resection are the most frequent sites of tumor recurrence, the normal parenchyma-tumor core interface (termed *tumor edge*) presumably contains postsurgical tumor-initiating cells (TICs; also termed recurrence-initiating cells) after craniotomy. Molecular and, more importantly, phenotypic characterization of these edge-TICs may lead to the identification of a means to inhibit the process of tumor recurrence after failure of the current therapies for glioblastoma.^16,17^

Diffuse infiltrative gliomas, when they recur, are detected by the propagation of new tumor core lesions, indicating the edge-to-core (E-to-C) transition is likely a critical step toward patient lethality. Nonetheless, these lethal seeds for tumor recurrence are mostly, if not entirely, surgically untouchable due to the presence of intermingled normal functional brain cells including neurons. In fact, despite recent advances in surgical technology increasing the extent of resection of the tumor *core* lesions to up to over 99%, the improvement of postsurgical patient survival surprisingly remains marginal. Therefore, further attention needs to be placed on the characterization and targeting of the remaining *edge* lesions (T2/FLAIR abnormality without Gadolinium enhancement on MRI), the cause of E-to-C progression and lethal tumor recurrence. In order to uncover the functional roles of tumor cells within this edge microenvironment, our recent clinical practice has undertaken an advanced program to isolate and characterize regionally distinct tumor cell populations by the supra-total resection during awake surgery to obtain reasonable amounts of edge tissues without harming patients. This has allowed us to functional identify *CD133* and *CD109* as the representative molecules of the *edge*-located and *acquired core*-associated TICs, respectively.^3,16,18,19^

In this study, we investigated this presumptive conversion of CD133^high^/CD109^low^ cells to CD133^low^/CD109^high^ cells as representative of highly lethal E-to-C dynamics by using 37 pairs of samples from matched primary and recurrent glioblastoma tumors. We then postulated that the decline of CD133 expressing TICs and the increase of CD109-expressing TICs indicates active E-to-C progression, worsening patient prognosis. To test this idea, we segregated our longitudinal sample set into 4 groups based on the CD133/109 expression changes. A set of integrated multimodal analyses was performed, followed by the preclinical validation of the identified molecular target as a functional key determinant for E-to-C-related glioblastoma aggressiveness.

## Materials and Methods

### Patients, Specimens, and Ethics

All 37 longitudinal glioblastoma cases were treated at Samsung Medical Center and Seoul National University Hospital and the tumor tissues were collected for research under the approved institutional review boards. Detailed methods are described in the previous study^16^ and Supplementary Material. For the preclinical studies, 4 patient-derived glioma sphere models were used, including 3 pair of tumor core- and edge-derived ones (1051E and C, 1053E and C, and 0573E and C) as well as one tumor edge-derived sphere line (101027E), which were established and described elsewhere.^3,16,18–22^ In short, with the signed patient consent, the senior author (I.N.) performed supra-total resection of glioblastoma tumors under the awake setting and resected both tumor core (T1-Gadolinium(+) tumors) and edge (T1-Gadolinium(-)/T2-FLAIR abnormal tumors in the noneloquent deep white matter) to achieve maximal tumor cell eradication without causing any permanent major deficits in the patients (Supplementary Figure 1A). After the confirmation of enough tumor tissues from both lesions secured for the clinical diagnosis, remaining tissues were provided to the corresponding scientists following de-identification of the patient information. Both the core-derived and edge-derived glioma spheres were established in the same culture condition^3,16,18–24^ and their spatial identities, termed *core-ness* and *edge-ness*, were confirmed by a set of xenografting experiments into mouse brains (details described in ref. ^16^). Only those that passed this confirmation were used in this study. The other patient-derived glioma sphere models without spatial information were established as “core-associated glioma spheres” using the same protocol and reported elsewhere.^16–23^ All these patient-derived glioma models were periodically checked with the mycoplasma test and the short tandem repeat analysis. All work related to preclinical data was performed under an Institutional Review Board-approved protocol (N150219008) compliant with guidelines set forth by National Institutes of Health (NIH).

### Public Microarray Data Processing

Three RNA sequencing datasets were downloaded from the Gene Expression Omnibus database (https://www.ncbi.nlm.nih.gov/geo/), including GSE63035, GSE67089, and GSE113149.^19,21,25^ RNA sequencing data of 29 longitudinal samples were derived from GSE63035, and 8 longitudinal samples were newly added; all the samples were IDH-wild type. The GSE67089 datasets contained gene expression data of MES, PN glioma sphere cells, and Neuron progenitor cells. The GSE113149 included the microarray data for sh-NT versus sh-CD109 in glioblastoma sphere 267. The RNA sequencing data of TCGA database were acquired from the TCGA Research Network (https://www.cancer.gov/tc-ga) and visualized by Gliovis^26^ (http://gliovis.bioinfo.cnio.es/).

### In Vitro Experiments

Detailed methods are described in the Supplementary Material.

### In Vivo Mouse Experiments

All animal experiments were performed under the Institutional Animal Care and Use Committee (IACUC)-approved protocol according to NIH guidelines. Detailed methods are described in the Supplementary Material.

### Statistical Analysis

All data are presented as mean ± SD. The number of replicates for each experiment was stated in Figure legends. Statistical differences between 2 groups were evaluated by 2-tailed *t*-test. The statistical significance of Kaplan–Meier survival plot was determined by log-rank analysis. A statistical correlation was performed to calculate the regression *R*^2^ value and Pearson’s correlation coefficient. Statistical analysis was performed by Prism 8 (GraphPad Software), unless mentioned otherwise in figure legend. *P* < .05 was considered as statistically significant.

## Results

### Patients in CD133^low^/CD109^high^ Group Exhibit Worse Prognoses With a Trend Toward an Increased Mesenchymal Signature

**Figure 1.**
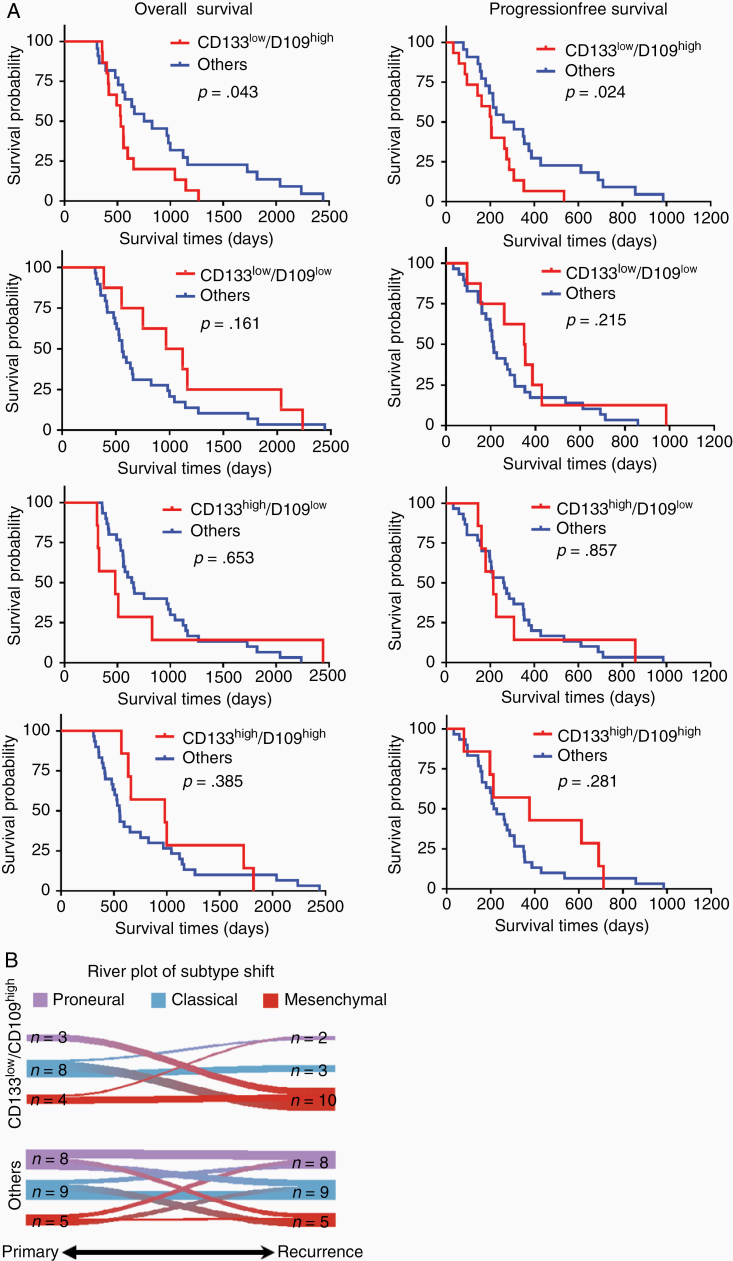
CD133^low^/CD109^high^ group exhibits worse prognoses accompanied by an increase in mesenchymal signature. (A) Kaplan–Meier analysis of overall survival (left) and progression-free survival (right) of glioblastoma patients in CD133^low^/CD109^low^, CD133^high^/CD109^low^, CD133^high^/CD109^high^, and CD133^low^/CD109^high^ (red, top to bottom) with each collected remaining cases (Others, blue). (Log-rank test). (B) River-plot analysis of the molecular subtype shifts from primary to recurrence in CD133^low^/CD109^high^ (upper) and others (lower) (*P* = .028, chi-square test).

To expand upon our previous studies,^16,19^ we used *CD133* and *CD109* mRNA expression profiles to indicate edge-ness and core-ness, respectively, a concept that we validated with 19 paired glioblastoma edge- and core-samples (Supplementary Figure 2). We reasoned that the loss of *CD133* mRNA (CD133^low^) and gain of *CD109* (CD109^high^) were indicative of the E-to-C progression in glioblastoma. Based on the differential RNA expression profiles as determined by RNA sequencing (seq) of 37 primary and recurrent glioblastoma pairs, 15 patients were assigned to the CD133^low^/CD109^high^ group as representative of E-to-C transition, while the other 22 patients were assigned as control arms (Others, either CD133^low^/CD109^high^, CD133^high^/CD109^low^, or CD133^high^/CD109^low^) for comparison. Both groups displayed similar average age, sex, distant recurrence profiles, and postsurgical therapy regimens (Table 1, Supplementary Table 1). We then investigated the progression-free survival and overall survival in these 4 groups. The CD133^low^/CD109^high^ group exhibited a substantially worse progression-free survival (*P* = .024) and overall survival (*P* = .043) compared with others (Figure 1A). Consistent with recent studies, both primary and recurrent tumors showed no significant difference in proportion among the 3 transcriptomic subtypes.^6^ However, there was a trend that CD133^low^/CD109^high^ group was enriched in tumors of the mesenchymal subtype upon recurrence (*P* = .028; Figure 1B). Importantly, in this patient cohort, the mesenchymal-ness of either primary or recurrent tumors did not show statistically significant differences in prognosis. These findings suggested a significant association between the CD133^low^/CD109^high^ signature representing E-to-C progression and poorer patient prognoses, associated moderately with increase of the mesenchymal subtype in the primary-to-recurrent glioblastoma progression.

**Table 1.** Demographics and Clinical Characteristics of the Patients in This Study

	CD133^low^/CD109^high^	Others	*P*-value
No. of patients	15	22	
Age	54.0 ± 10.2	48.2 ± 9.3	NS
Sex (M/F)	8/7	13/9	NS
% of distant recurrence	33.3% (5/15)	27.3% (6/22)	NS
Radiotherapy between first and second surgery (%)	100% (15/15)	95.5% (21/22）	NS
Chemotherapy between first and second surgery (%)	80% (12/15)	95.5% (21/22)	NS
Delta-CD133	−1.19 ± 0.98	0.21 ± 0.85	5.02E−5
Delta-CD109	0.93 ± 0.76	−0.20 ± 0.77	9.58E−5

NS, not significant.

### Longitudinal RNA-seq Analysis Identifies the Differential Expression Profile Associated With E-to-C Including PLAGL1 and CD109

**Figure 2.**
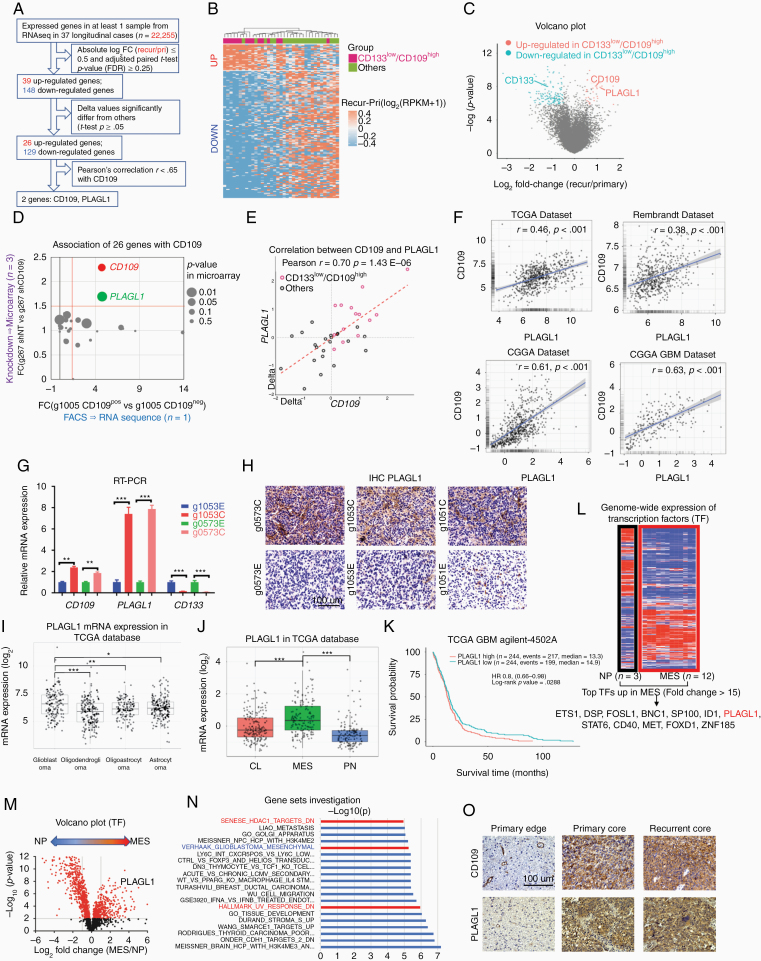
Longitudinal RNA-seq analysis identifies the differential expression profile associated with ECT including *PLAGL1* and *CD109*. (A) Schematic demonstration of the filtering procedure of *PLAGL1* from 22,255 genes. (B) Heatmap depicting supervised hierarchical clusters of up- and down-regulated genes within recurrent glioblastomas of CD133^low^/CD109^high^ and others. (C) Volcano plot of RNA-seq data comparing CD133^low^/CD109^high^ and others. Red and blue dots refer to up- and down-regulated genes in CD133^low^/CD109^high^ group, respectively. (D) Scatterplot comparing expression profiles of the 26 genes from RNA-seq analysis results within our tumor core-derived glioma sphere models; g1005 (*n* = 1) FACS-sorted into CD109 negative to positive cells and represented along the x-axis (left to right, respectively), while microarray relative expression of *CD109* in g267 (*n* = 3) with shNT and shCD109 is represented along the y-axis (down- and up-ward, respectively). (E) Scatterplot displaying the linear correlation between *CD109* and *PLAGL1* expressions in the 37 longitudinal cases. Pearson correlation coefficient (*r*) = 0.70 and *P* = 1.43E−06. (F) Scatterplot displaying the linear correlation between *CD109* and *PLAGL1* expressions in 4 public databases (TCGA, Rembrandt, CGGA, and CGGA GBM), based on Pearson correlation test. (G) Bar graph displaying qRT-PCR results for the expression of *CD109, PLAGL1*, and *CD133* within edge- and core-derived sphere culture models of 2 glioblastoma patients (g1053 and g0573). Data are means ± SD (*n* = 3). ***P < .001. (H) Representative images of immunohistochemistry (IHC) for PLAGL1 in mouse orthotopic xenografts with tumor core- (C) and edge(E)-derived glioma sphere models from 3 patients (g0573, g1053, and g1051). Scale bar 100 µm. (I) Boxplot diagram demonstrating *PLAGL1* relative mRNA expression profiles from TCGA database across different gliomas subtypes. *P < .05, **P < .01, and ***P < .001. (J) Boxplot diagram comparing relative expression profiles of *PLAGL1* among the 3 molecular subtypes (classical, mesenchymal [MES], and proneural) of glioblastoma within TCGA database. ***P < .001. (K) Kaplan–Meier survival curve of glioblastoma patients in the TCGA database. Patients were categorized into a “high” or “low” expression group based on the median *PLAGL1* expression in the Agilent 4502 microarray. (L) Heatmap of displaying expression profiles of transcription factors (TFs) (*n* = 2,766) across 4 MES glioma sphere lines compared with the neural progenitor sphere line (NP) (*n* = 3 for each cell line). (M) Volcano plot comparing TF gene expressions (*n* = 2,766) across MES and NP lines, highlighting *PLAGL1*. (N) Upregulated pathways in recurrent glioblastomas in CD133^low^/CD109^high^ group. (O) Representative IHC images for CD109 and PLAGL1 in primary edge and core, and recurrent core tumor tissues. Scale bar 100 µm.

Next, we pursued a stepwise approach to identify a molecular target or targets that could mediate the observed molecular and phenotypic E-to-C dynamics. First, we established a data analysis pipeline using all expressed genes in the RNA-seq data of the 37 longitudinal cases (*n* = 22,255; Figure 2A). Differential gene expression analysis identified 26 genes distinctively associated with the CD133^low^/CD109^high^ changes (Supplementary Table 2). Unsupervised hierarchical clustering of those genes (*n* = 155) segregated our cohort sample (*n* = 37) into 2 distinctive subgroups (up- and down-regulated) (Figure 2B and C). In order to further elucidate the E-to-C-associated essential molecules, we designed an integrated second step approach to evaluate the expression of these 26 upregulated genes in our well-characterized glioma sphere models treated with either shRNA-based gene silencing of *CD109* or flow cytometry to isolate CD109(+) cells. To this end, we used our recently published RNA-seq data with 2 well-characterized tumor core-derived glioma sphere models; g267 for shRNA and g1005 for flow cytometry.^19^ As a result, *PLAGL1* was identified as being the gene whose expression most strongly correlated with that of *CD109* (FC>1.5, *P* < .05; Figure 2A and D, Supplementary Figure 3A and B). Consistently, Pearson’s correlation analysis of the 37 glioblastoma paired samples indicated a strong linear relationship between *CD109* and *PLAGL1* relative expression (*r* = 0.7, *P* < .05; Figure 2E). This *CD109-PLAGL1* expression correlation was also observed in 4 clinical datasets (TCGA, Rembrandt, CGGA, and CGGA GBM datasets; Figure 2F). qRT-PCR with 2 additional edge- and core-derived glioma sphere models (Edge- and Core-derived g1053 spheres and g0573 spheres) showed that both *PLAGL1* and *CD109* were higher in the core-derived, yet *CD133* was up in the edge-derived, glioma spheres in vitro (Figure 2G).

To prospectively assess PLAGL1 localization in experimental tumors, we injected edge- or core-derived glioma spheres from 3 patients into immunodeficient mice. PLAGL1 showed its preferential expression in the tumor core-derived lesions (Figure 2H). In the patient tumor data in TCGA, *PLAGL1* mRNA expression was markedly elevated in glioblastomas compared to lower grade gliomas (Figure 2I). In glioblastoma, *PLAGL1* mRNA was relatively higher in mesenchymal tumors (Figure 2J). As expected, glioblastoma patients with higher *PLAGL1* expression exhibited shorter survival in the TCGA database (Figure 2K).

Since the *PLAGL1* gene encodes for C2H2 zinc finger (ZF) transcription factors (TFs),^27^ we sought to further confirm our results by cross-referencing them to our previously established cDNA microarray dataset with the sphere lines established from either human neonatal brains (neural progenitors [NPs]) or glioma patients with mesenchymal or core-associated signature.^21^ Among 2,766 human TFs,^28^ 12 TFs, including *PLAGL1*, were highly overexpressed (fold change >15) in mesenchymal or core-associated glioma sphere lines as opposed to NP counterparts (*P* < .001; Figure 2L), moreover, *PLAGL1* was the second highest C2H2-ZF TFs in mesenchymal tumor cells (Supplementary Figure 4). Consistently, a volcano plot displayed *PLAGL1* as a significantly upregulated gene in mesenchymal or core-associated glioma spheres (Figure 4M). Gene set enrichment analysis (GSEA) using the 26 upregulated genes identified their correlation with “*HDAC1 targets*” and “*UV response DNA damage*,” both of which our recent studies have identified as pathways downstream of the CD109-driven signals in glioblastoma tumors and their TIC models (Figure 4N).^16,19^ Finally, we explored the expression of PLAGL1 in primary glioblastoma edge, core lesions as well as their subsequent recurrent core tissues, which showed PLAGL1 higher in the core lesions in both primary and recurrent tumors (Figure 4O). Collectively, these clinical and experimental data suggested PLAGL1 is one key regulator in the edge-TICs to cause tumor core development in glioblastoma.

### Genetic Perturbation of PLAGL1 Reveals Its Role in Glioblastoma Tumorgenicity in the Edge-TIC Models

**Figure 3.**
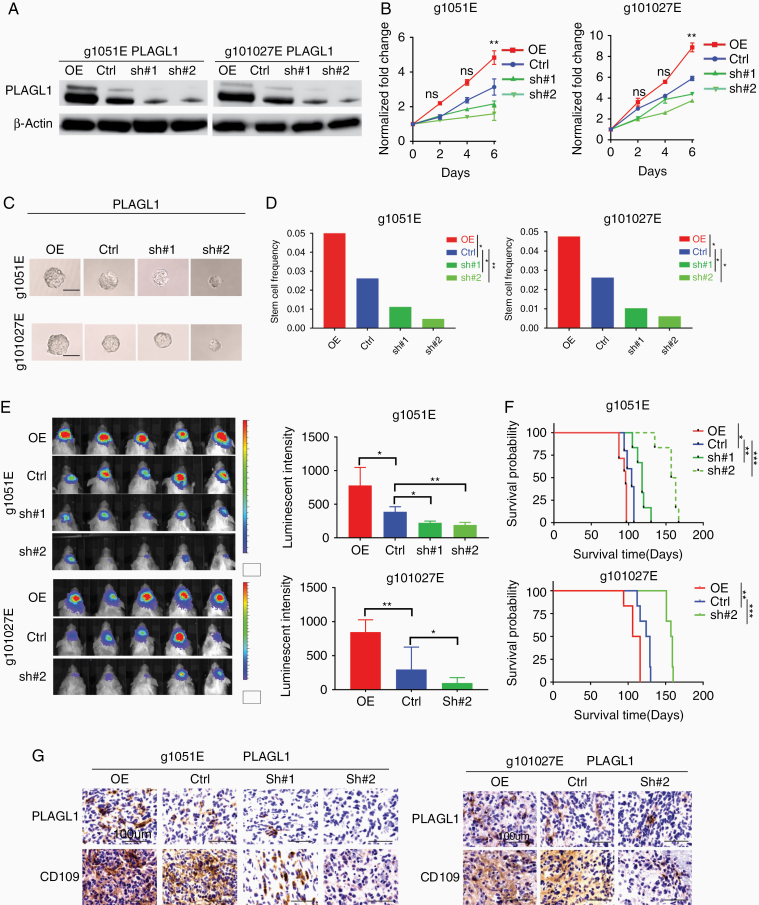
PLAGL1 overexpression enhances, while its silencing diminished, glioblastoma growth in vivo, leading to prolong mouse survival in the edge-TIC models. (A) Western blotting of 2 patients’ tumor edge-derived glioma sphere lines (g1051E and g101027E) after transducing with overexpression vector (OE) or shRNA targeting *PLAGL1* (sh#1 or sh#2) or a nontargeting control (Ctrl). (B) Line charts of in vitro growth of the indicated groups (**P < .01, *n* = 6, 1-way ANOVA). (C) Representative images of the indicated glioma sphere lines after genetic transduction. Scale bar 60 μm. (D) Bar graphs of the limiting dilution glioma sphere forming assay, depicting the relationship between PLAGL1 expression and clonal populations in the edge-derived glioma spheres (g1051E, g101027E) (*P < .05, **P < .01, ELDA analyses). (E) Bioluminescent images (left) and their quantifications (right) of orthotopic mouse xenografts established by injection of indicated g1051E and g101027E glioma sphere models (*P < .05 and **P < .01, *n* = 5, 1-way ANOVA). (F) Kaplan–Meier analysis of immunocompromised mice harboring intracranial tumors derived from g1051E or 101027E spheres transduced with either overexpressed PLAGL1 (*n* = 7), Ctrl (*n* = 5), shPLAGL1#1 (*n* = 6), or shPLAGL1#2 (*n* = 6). *P < .05, **P < .01, and ***P < .001. (G) IHC of indicated tumors in immunocompromised mice for CD109 and PLAGL1. Scale bar 100 µm.

Following the identification of PLAGL1 as a potential candidate regulating the E-to-C-mediated glioblastoma malignancy, we investigated the function of PLAGL1 in our glioblastoma edge-TIC models to understand if its targeting holds any translational significance. To this end, we used the 2 tumor edge-derived glioma sphere models (1051E and 101027E) for lentivirus-mediated gene overexpression (PLAGL1-OE) and knockdown by shRNA (sh#1 and #2). As the control, we used the nontargeting lentiviral construct (Ctrl). Western blotting confirmed both induced overexpression and gene silencing in cells harboring the shRNA construct, with more efficient targeting of PLAGL1 by sh#2 than sh#1 (Figure 3A, Supplementary Figure 5A). In both models, PLAGL1-OE displayed significantly higher in vitro growth rates, while their growth was largely attenuated by gene silencing of PLAGL1 (Figure 3B). Using clonal sphere formation as a surrogate in vitro indicator of tumor initiating capacity, we found that PLAGL1-OE glioma spheres relatively increased, whereas its gene silencing reduced it with a greater inhibitory effect of sh#2 compared to sh#1 (Figure 3C and D, Supplementary Figure 5B). In vivo injection of PLAGL1-OE glioma spheres into brains of immunocompromised mice resulted in higher luminescent intensity indicative of their larger tumor sizes by edge-TIC-derived tumor establishment, whereas the shRNA-carrying xenografts displayed significantly lower signals in both of these 2 glioblastoma edge sphere-derived tumor models (Figure 3E). Mice with PLAGL1-OE glioma sphere-derived tumors exhibited significantly worse survival due to higher tumor burden, while their gene silencing groups displayed improved overall survival by lower tumor burden compared to the control group (Figure 3F, Supplementary Figure 6). As expected, immunoreactivity to CD109 was strongly correlated with the expression of PLAGL1 in both models (Figure 3G). Collectively, these data suggested that PLAGL1 regulates the in vitro clonality and in vivo tumor development originally derived from edge-TICs in glioblastoma.

### PLAGL1 Binds to the Promoter Region for CD109 to Regulate Its Transcriptional Activity

**Figure 4.**
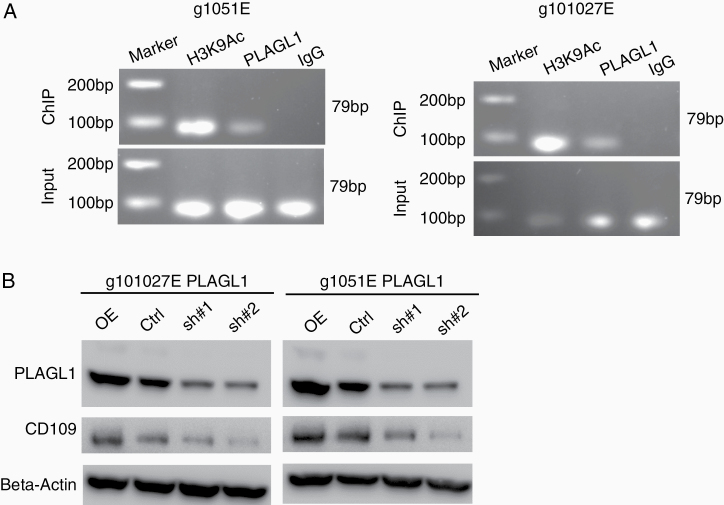
PLAGL1 binds to the promoter region for CD109 to positively regulate its transcriptional activity. (A) Chromosome Immunoprecipitation (ChIP)-qPCR assay showing PLAGL1 binding to the promoter region for the *CD109* gene in g1051E and g101027E spheres. H3K9Ac is used as a positive control. (B) Western blotting of CD109 and PLAGL1 in g1051E and g101027E spheres after transducing with overexpression vector, shRNA targeting *PLAGL1* (sh#1 or sh#2), or a nontargeting control (Ctrl).

Lastly, we sought to determine the molecular mechanisms connecting PLAGL1 and CD109. Specifically, we tested if PLAGL1 as a TF binds to the promoter region for the CD109 gene in glioblastoma edge-derived cells. Using g1051E spheres, we performed chromatin-immunoprecipitation with the PLAGL1 antibody, followed by qRT-PCR for the *CD109* genetic regulatory element that we previously identified as its active promoter.^19^ A band was detected that is indicative of the direct transcriptional regulation of *CD109* by PLAGL1. This result was also validated with g101027E cells (Figure 4A). As expected, overexpression of PLAGL1 elevates, while its silencing decreases, the expression of CD109 protein, determined by western blotting in both sphere models (Figure 4B). Collectively, the tightly associated co-expression of PLAGL1-CD109 was, at least in part, mediated through the direct transcriptional regulation of CD109 via the TF, PLAGL1.

## Discussion

**Figure 5.**
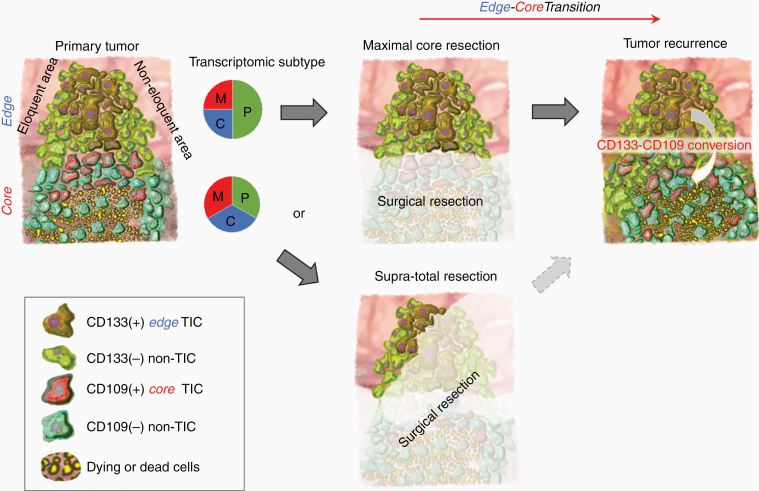
Schematic delineating the edge- and core-located tumor cells in glioblastoma together with intratumoral CD133 and CD109 expressions in the TIC subpopulations.

Patients with glioblastoma gain only limited benefit from craniotomy due to inability to completely eliminate tumor cells from the brain.^29,30^ The lethal seeds for tumor recurrence (*recurrence-initiating cells*) reside predominantly, yet not entirely, at the tumor edge surrounding the resection cavity. In this study, we used *CD133* and *CD109* mRNA expression dynamics as a reference to indicate the E-to-C progression in glioblastoma. The rationale for this investigation included our previous findings that both *CD133* and *CD109* are preferentially expressed within the TIC subpopulations, selectively within glioblastoma edge- and core-tissues, respectively.^19^ While the expression of *CD109* and *CD133* within individual cells in tumors appears to be mutually exclusive, the expression of these markers appears to represent a dynamic molecular state.^16,19^ One means of promoting E-to-C transition is through radiation, which induces the conversion of edge-associated CD133(+)/CD109(-) TICs to the core-associated CD133(-)/CD109(+) TICs, thereby developing therapy-resistant tumors in vivo.^19^ On the other hand, core TICs themselves respond to radiation by secreting factors that promote the radio-resistance of edge-located TICs in vitro and in vivo.^16^ Furthermore, core- and edge-TICs are dependent on distinct metabolic and kinomic pathways for their survival and growth.^18,31^ Thus, only by targeting both core- and edge-TICs would presumably enable to achieve better outcomes of glioblastoma patients. However, recent advances in surgical technique, including the imaging-assisted fluorescence-guided surgery in the awake setting, have allowed us to increase the proportion of surgical cases of total or near-total resection of the tumor core lesions. Yet, even with the attempt for supra-total resection of glioblastoma tumors (Figure 5), edge-located tumor cells undoubtedly remain, where the recurrence-initiating cells are hidden.

In this study, we used 37 paired primary-recurrent glioblastoma samples—the largest matched cohort published thus far—to examine E-to-C transition, resulting in validation of its association with poorer patient prognoses. It is important to note that both tumor edge and core are composed of tumor cells in all 3 transcriptomic subtypes, albeit the ratios are slightly different (Figure 5).^16^ Our findings suggest, yet do not definitely prove, relatively weaker correlation of mesenchymal-ness, in comparison to the E-to-C signature, to patients’ poorer prognosis, at least in this patient cohort. This interpretation needs further validation with more clinical evidence, ideally with prospective measurement, from multiple independent groups. The E-to-C axis could be more clinically relevant but it remains poorly understood how similar to, or different from, the transcriptomic proneural (and classical)-mesenchymal axis it is. In addition, in some craniotomies, small residual core lesions are inevitably unresectable by surgery, depending on the proximity/invasion to functional brain areas. There is no doubt that these core tumor cells also contribute both directly and indirectly to glioblastoma recurrence, as our recent study suggested.^16^ Therefore, we need to be cautious that E-to-C progression does not explain all the clinical cases of the primary-to-recurrent glioblastoma progression. More extensive molecular characterization with additional longitudinal case cohorts is warranted.

For the phenotypic characterization associated with tumor edge and core in glioblastoma, we believe that the recently established tumor edge- and core-derived glioma spheres represent valuable models, as their xenografts faithfully recapitulate their spatially distinct tumor lesions in mouse brains.^3,16,18,31,32^ These models enable the investigation of glioblastoma recurrence from post-surgical residual diseases with varying populations of the mixed tumor edge and core cells. Needless to say, the accuracy and validation of the resected tissue locations within the brain is critical for these spatially identified models and a number of potential hurdles have to be overcome in order to ascertain their reliability (eg, brain shift, patient safety). In particular, glioblastomas tend to infiltrate into the deep white matter, where a number of functional neuronal fibers run throughout the brain. Obtaining tumor edge tissues from these regions without harming the patients is a critical step for surgeons to cooperate with scientists to establish models that faithfully recapitulate their spatially distinct pathobiology. Further characterization of our models and developing other tumor edge-reflective models would help facilitate the molecular and phenotypic analyses to identify therapeutic targets in the recurrence-initiating cells at tumor edge that subsequently cause patient lethality.

Regarding the molecular mechanism, our data indicated the significance of targeting PLAGL1 to attenuate, yet not completely eliminate, tumor initiation and propagation, accompanied by an impact on survival of tumor-bearing mice. This TF directly regulates the E-to-C-associated gene *CD109* in tumor edge-derived cells. CD109 was previously found to drive E-to-C transition,^16,19^ and, thus, by inference, PLAGL1 would be expected to do the same. Nonetheless, the role of PLAGL1 in cancer has been controversial. Prior studies have shown that PLAGL1 is a tumor suppressor gene encoding an inducer of apoptosis and cell cycle arrest in various cancers^33–35^ (eg, breast cancer, hepatoma, and colon cancer). Even in glioma, one study has demonstrated the frequent loss of PLAGL1 in their clinical samples.^36^ On the other hand, another study paradoxically demonstrated a pro-tumorigenic function of PLAGL1 driven by SOX11.^37^ Here, using the preclinical models, we provide a set of experimental evidence to support the tumorigenic function of PLAGL1 in glioblastoma TICs. In addition, the analysis of clinical samples using public and our own databases supported our experimental findings. The PLAGL1-mediated signaling might be context dependent among various cancer cells, or even within gliomas. We previously found that HDAC1 is a positive transcriptional regulator that drives CD109 gene expression via a protein complex formation with an oncogenic TF C/EBPβ,^16^ even though HDAC1 is recognized to modulate the compact chromatin structure leading to widespread repression of transcriptional activities in cancers and developmental somatic cells.^38–40^ It remains unknown if PLAGL1 forms a larger protein complex with HDAC1 and C/EBPβ in glioblastoma and other cancers. In addition, the upstream regulator(s) for the PLAGL1 signaling in the E-to-C process needs further investigation. Given that microenvironmental cues take critical roles particularly at the tumor edge (eg, neuronal BDNF^41^ and vascular endothelium-derived endocan^17^), both extra-cellular and cell-intrinsic signals may cooperatively orchestrate the PLAGL1-driven activation of the E-to-C progression in tumors. As the molecular and cellular complexity of glioblastoma is increasingly recognized as a challenging road block to prolong survival of patients, successful removal of tumor core is certainly the mandated first step, yet it still requires us to learn how to manage the tumor edge in better ways. Further phenotypic characterization of edge-located recurrence-initiating cells is among key tasks ahead of us to develop effective therapies for glioblastoma.

## Supplementary Material

vdaa163_suppl_Supplementary_Figures

vdaa163_suppl_Supplementary_Figure_Legend

vdaa163_suppl_Supplementary_Materials

vdaa163_suppl_Supplementary_Table_S1

vdaa163_suppl_Supplementary_Table_S2

vdaa163_suppl_Supplementary_Table_S3

vdaa163_suppl_Supplementary_Table_S4

## Notes

**Authorship Statement.** Leading conceptualization of the study: I.N. Main financial support: D.H.N. and I.N. Laboratory practice: C.L., Q.C., S.Y., S.K., and S.G. Bioinformatic analysis: C.L., H.J.C., D.Y., D.H.N., and I.N. Schematic drawing of hypothesis (Figure 5): I.N. Drafting the article: C.L. and I.N. Critical revision of the article: H.I.K., D.H.N., and I.N. All authors had substantial input to the logistics of the work and revised and approved the final manuscript. The authors know their accountability for all aspects of the study ensuring that questions regarding the accuracy and integrity of any part are appropriately investigated and resolved. The corresponding authors had full access to all of the data and the final responsibility to submit the publication.
