# Supplementary material for: Tumor edge-to-core transition promotes malignancy in primary-to-recurrent glioblastoma progression in a PLAGL1/CD109-mediated mechanism
Source: Neurooncol Adv. 2020 Nov 27;2(1):vdaa163. doi: 10.1093/noajnl/vdaa163 (PMC7764499; doi:10.1093/noajnl/vdaa163)

Supplementary Figure.1

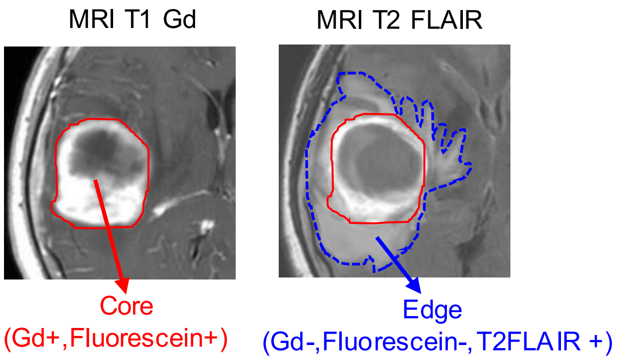

Supplementary Figure.2

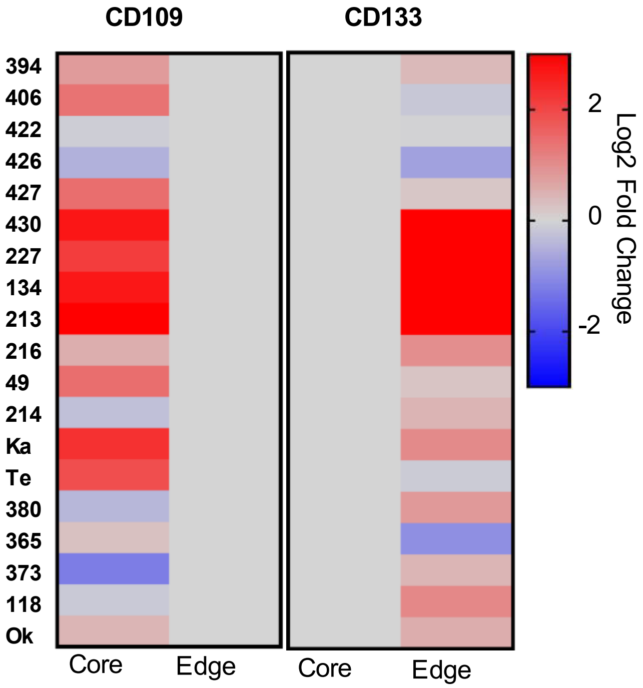

Supplementary Figure.3

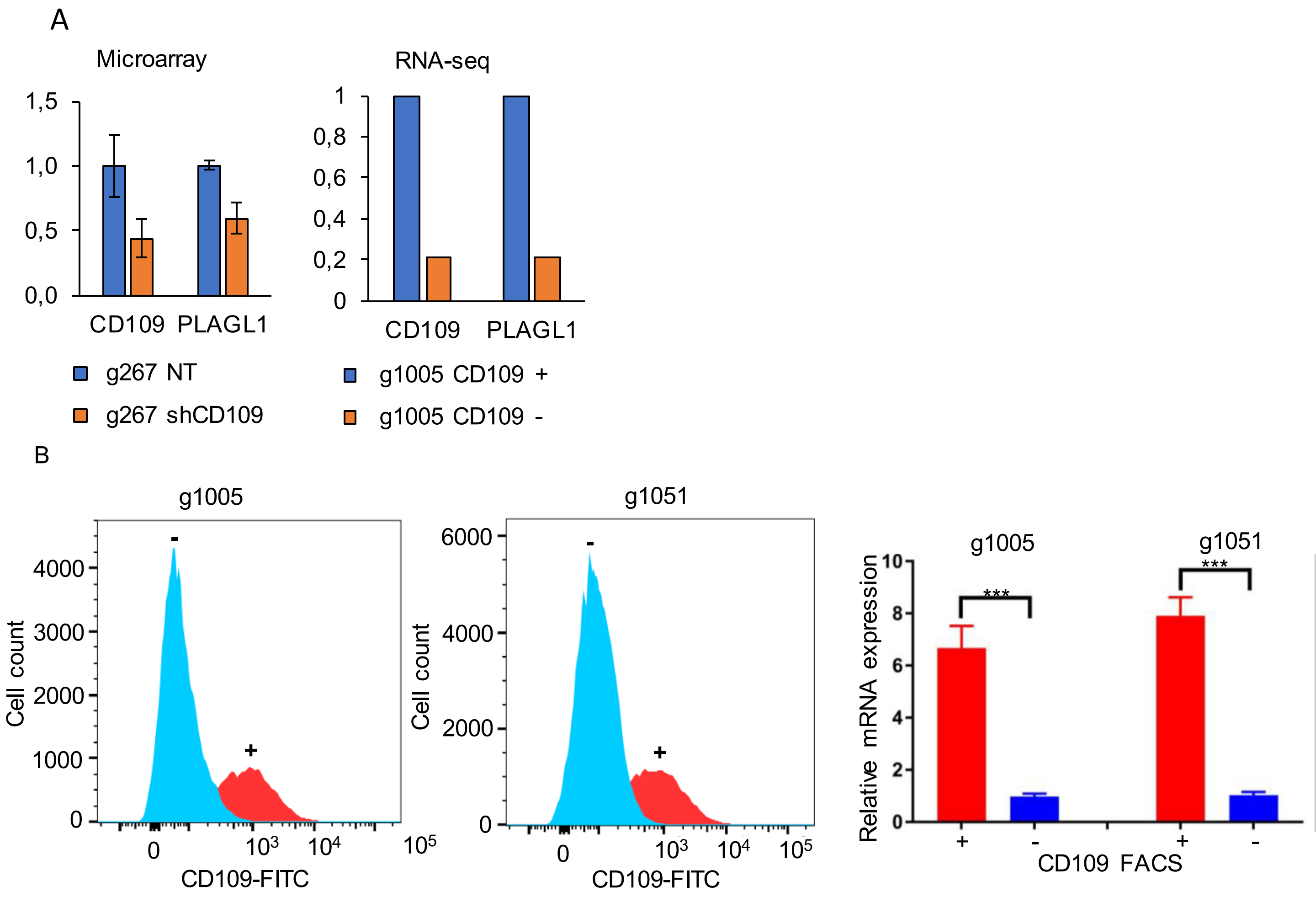

Supplementary Figure.4

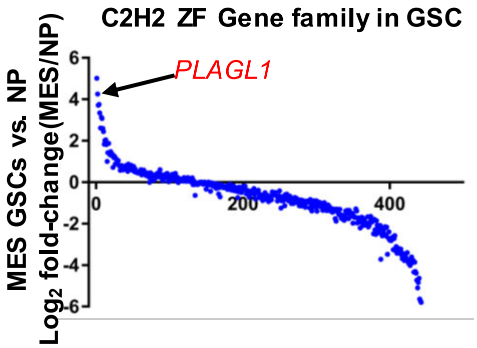

Supplementary Figure.5

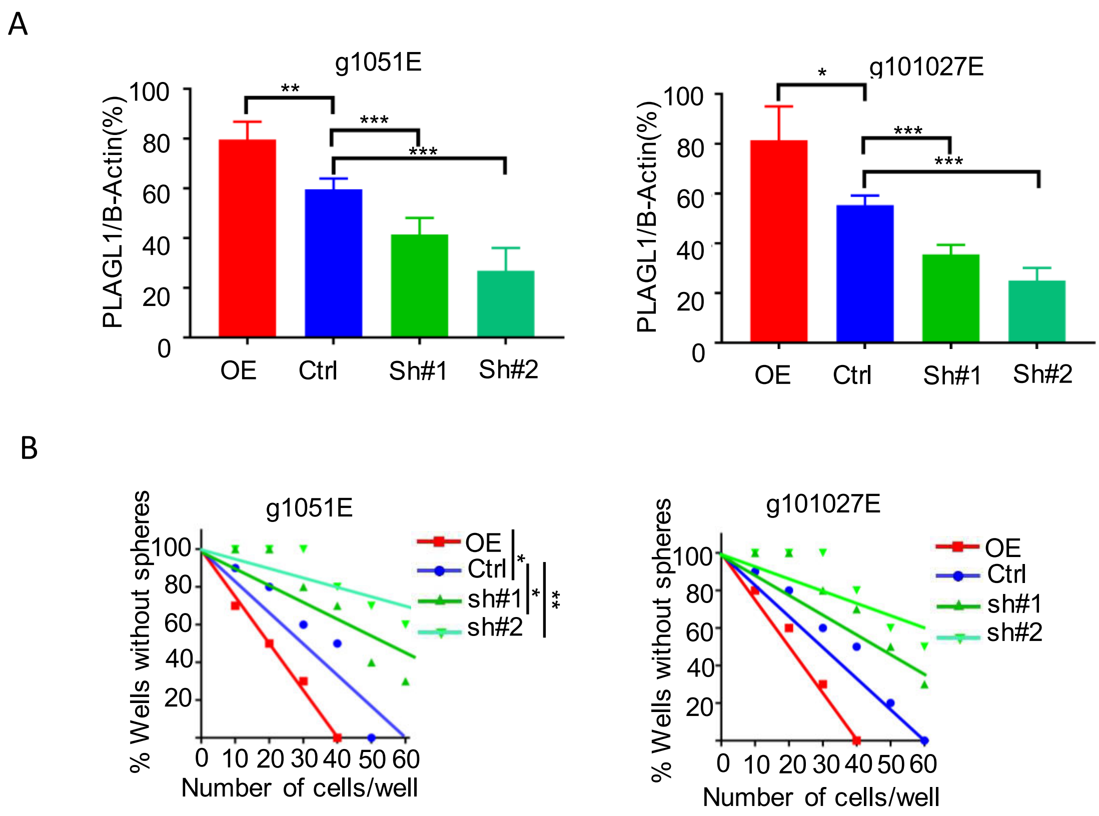

Supplementary Figure.6

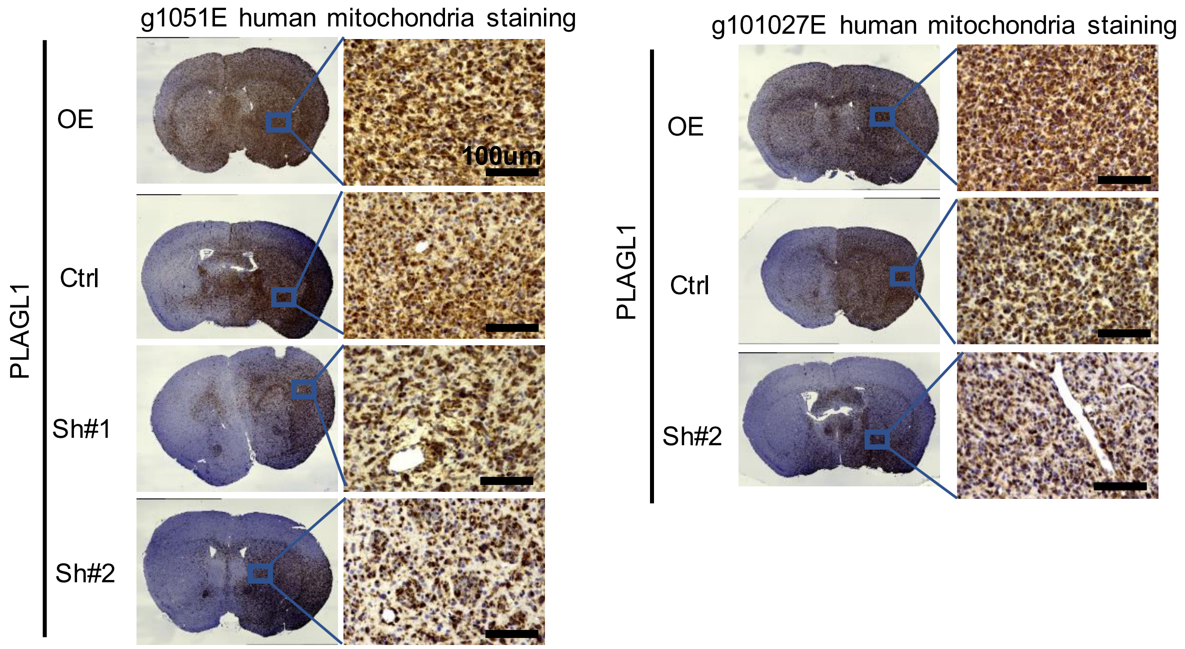

Supplementary Figure.7

A

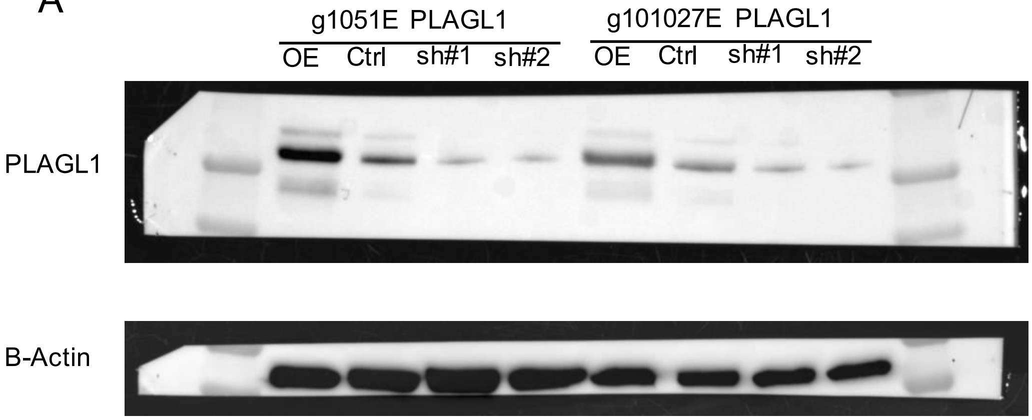

B

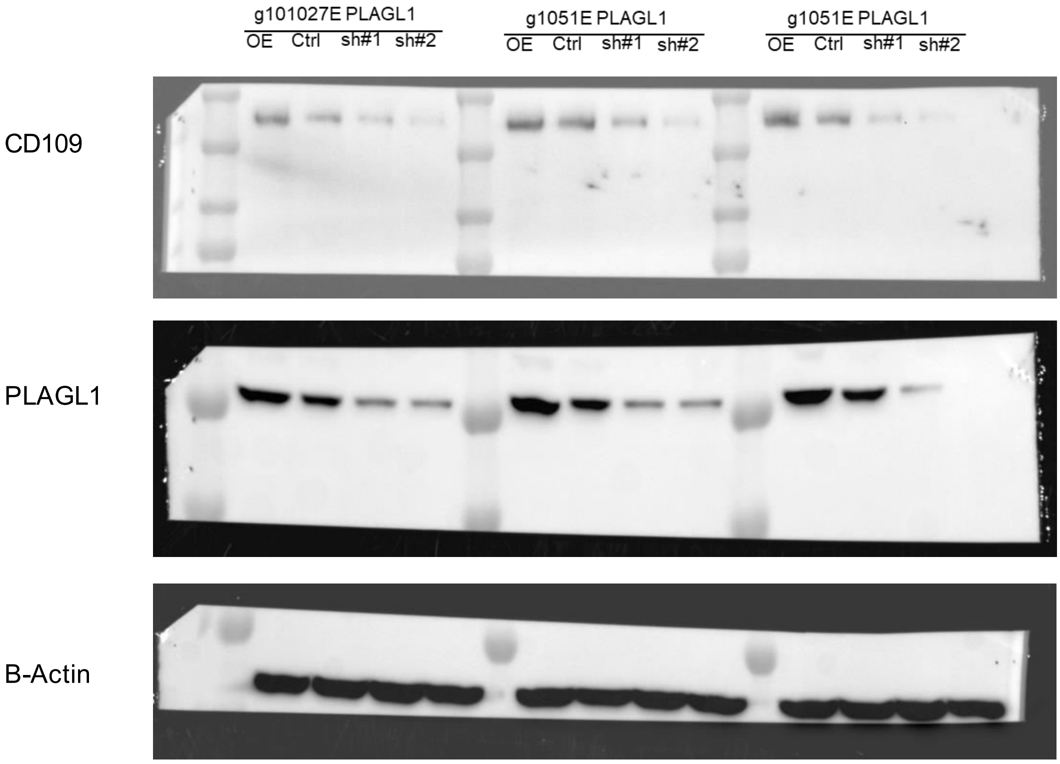

Supplement: vdaa163_suppl_Supplementary_Figures [file vdaa163_suppl_supplementary_figures.pdf]
