# Supplementary material for: Tumor edge-to-core transition promotes malignancy in primary-to-recurrent glioblastoma progression in a PLAGL1/CD109-mediated mechanism
Source: Neurooncol Adv. 2020 Nov 27;2(1):vdaa163. doi: 10.1093/noajnl/vdaa163 (PMC7764499; doi:10.1093/noajnl/vdaa163)
Supplement: vdaa163_suppl_Supplementary_Table_S3 [file vdaa163_suppl_supplementary_table_s3.docx]

| **Name** | **Sequence** |
| --- | --- |
| PLAGL1 FW | AAAGATGCTTCTACACCCGGA |
| PLAGL1 RV | AGTGGGTCTTCTTGGTATGCC |
| CD109 FW | AAGCCAGTGAAAGGAGACGTA |
| CD109 RV | CCAGGGGAAGATAGATCCAGG |
| CD133 FW | AGTCGGAAACTGGCAGATAGC |
| CD133 RV | GGTAGTGTTGTACTGGGCCAAT |
| GAPDH FW | GAAGGTGAAGGTCGGAGTCA |
| GAPDH RV | TTGAGGTCAATGAAGGGGTC |
|  |  |
| CD109 ChIP FW | CAGTGCGAGTTCTCTTCTTCTT |
| CD109 ChIP RV | GAGGTCTACTGCTTTCCTTTCC |

Table.3 List of primers used in this study.
